# Optimized Design of Direct Digital Frequency Synthesizer Based on Hermite Interpolation

**DOI:** 10.3390/s24196285

**Published:** 2024-09-28

**Authors:** Kunpeng Zhou, Qiaoyu Xu, Tianle Zhang

**Affiliations:** College of Mechanical and Electrical Engineering, Henan University of Science and Technology, Luoyang 471000, China; zkp@stu.haust.edu.cn (K.Z.); ztl@stu.haust.edu.cn (T.Z.)

**Keywords:** direct digital frequency synthesis, hermite interpolation, FPGA, amplitude quantization

## Abstract

To address the issue of suboptimal spectral purity in Direct Digital Frequency Synthesis (DDFS) within resource-constrained environments, this paper proposes an optimized DDFS technique based on cubic Hermite interpolation. Initially, a DDFS hardware architecture is implemented on a Field-Programmable Gate Array (FPGA); subsequently, essential interpolation parameters are extracted by combining the derivative relations of sine and cosine functions with a dual-port Read-Only Memory (ROM) structure using the cubic Hermite interpolation method to reconstruct high-fidelity target waveforms. This approach effectively mitigates spurious issues caused by amplitude quantization during the DDFS digitalization process while reducing data node storage units. Moreover, this paper introduces single-quadrant ROM compression technology to further diminish the required storage space. Experimental results indicate that, compared to traditional DDFS methods, the optimization scheme proposed in this work achieves a ROM resource compression ratio of 1792:1 and a 14-bit output Spurious-Free Dynamic Range (SFDR) of −88.134 dBc, effectively enhancing amplitude quantization precision and significantly lowering spurious levels. This significantly improves amplitude quantization precision and reduces spurious levels. The proposed scheme demonstrates notable advantages in both spectral performance and resource utilization efficiency, making it highly suitable for resource-constrained embedded systems and high-performance applications such as radar and communication systems.

## 1. Introduction

Amid the rapid advancement of electronic device technology, the demand for high-frequency and high-precision signals continues to grow [1]. Frequency synthesis technology is widely used to generate frequency sources in electronic devices, with four main types currently in use: Analog Direct Frequency Synthesis (ADS), Phase-Locked Loop (PLL), Pulse Output DDS (PDDS), and Direct Digital Frequency Synthesis (DDFS). ADS and PLL technologies, however, rely on analog implementation, which requires numerous analog components, leading to complex circuitry, bulky size, and excessive spurious signals. While PDDS is implemented using more compact standard cells, its 1-bit output structure limits its ability to produce high frequencies and achieve a high Spurious-Free Dynamic Range (SFDR). DDFS has become a prominent research topic due to its superior frequency resolution, fast frequency switching capabilities, and wide output range. Nonetheless, the performance of DDFS, particularly its SFDR, is significantly constrained by errors caused by phase truncation due to the limited ROM size [2,3,4].

To address the spurious issues in DDFS, current research commonly employs interpolation or dithering schemes. Tang et al. [2] proposed a piecewise linear approximation method that reduces spurious signals caused by quantization errors. While this approach increases the amplitude quantization level to minimize quantization errors, it also requires more complex parameter calculations, resulting in higher ROM resource consumption. Choi et al. [3] designed a novel low-power DDFS topology that utilizes dithering techniques to improve SFDR, enhancing signal quality while reducing system power consumption. However, this method introduces additional background noise, which may impact the overall signal-to-noise ratio (SNR) performance. Several studies have also explored ROM-less DDFS designs, using various algorithms instead of ROM tables to implement the phase-to-sine mapping function. Although these approaches eliminate ROM storage and reduce chip area, they introduce high computational complexity, leading to increased power consumption and resource usage [4,5,6]. Furthermore, implementing polynomials of orders higher than three has been found impractical in terms of hardware feasibility [5]. Currently, no method can achieve high SFDR without increasing ROM resource consumption, a critical factor in DDFS design and implementation.

To address these challenges, this paper proposes a DDFS design scheme based on the cubic Hermite interpolation algorithm. The key innovations of this design include:

1. Utilizing the smooth characteristics of cubic Hermite interpolation to effectively address waveform spurs caused by accumulator truncation. A dual-port ROM structure is employed to obtain the parameters required for interpolation calculations.
2. By leveraging the derivative relationship between sine and cosine functions in the ROM table design, this approach avoids excessive ROM resource consumption. Additionally, a single-quadrant ROM compression method is introduced to further reduce ROM size.

Although this design requires significant FPGA logic resources and multipliers, their usage does not increase exponentially with bit width, unlike ROM. This scheme meets higher spectral purity requirements while reducing ROM resources, offering a promising new approach for the design of efficient DDFS systems.

## 2. DDFS Architectural Design

### 2.1. Traditional DDFS Architecture

**Figure 1:**
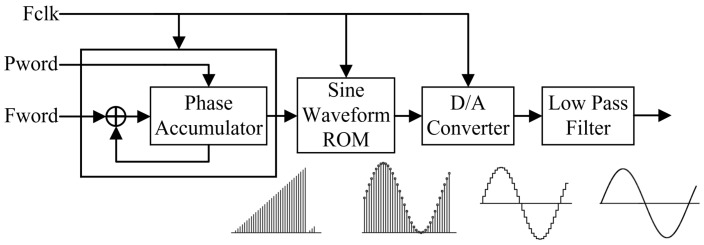
Block diagram of traditional DDFS structure.

In the traditional DDFS system architecture, which includes a phase accumulator, waveform lookup table, digital-to-analog converter (DAC), and low-pass filter (LPF) [7,8,9], as illustrated in Figure 1, the input data consists of a phase control word (Pword) and a frequency control word (Fword). With each system clock cycle, $f_{clk}$, the phase register performs an accumulation calculation, using Fword as the increment and combining it with Pword to determine the precise phase information of the waveform at that moment [10]. In this process, the phase data must be truncated and used as the index address for the ROM lookup table. Each address in the lookup table corresponds to an approximate phase point within the 0 to 2π range. The lookup table maps this address information to sine amplitude values, which are then output through the DAC and LPF to generate a smooth waveform signal [11].

**Figure 2:**
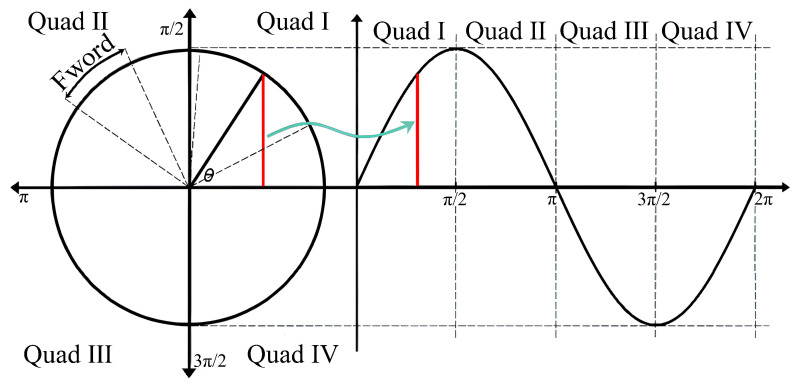
DDFS phase–waveform mapping relationships.

When the frequency register in a traditional DDFS is set to *N* bits and incremented by steps of Fword, the frequency register resets after every $2^{N}/Fword$ system clock cycle. Simultaneously, the sine lookup table address also returns to its initial value after completing a cycle, allowing the entire DDFS system to output a complete sine wave [3,12,13,14,15], as shown in Figure 2. The output frequency $f_{out}$ can be calculated as follows:

(1) $$fout=\frac{Fword}{2^{N}}fclk$$

In a traditional DDFS, the output signal fout is sampled at discrete intervals. The frequency spectrum A(ω) of this output signal fout can be expressed as follows [16]:

(2) $$A\left(\omega \right)=e^{-j\omega T/2}\cdot sinc\left(\frac{\omega T}{2}\right)\sum_{n=-\infty }^{+\infty }\delta \left[\omega -\left(\frac{2\pi n}{T}\pm \omega_{out}\right)\right]$$

where *T* is the sampling period; $sinc\left(\frac{\omega T}{2}\right)$ is the envelope of the spectrum; $e^{-j\omega T/2}$ represents the phase shift of the output signal; and $\sum_{n=-\infty }^{+\infty }\delta \left(\cdot \right)$ represents the summation of Dirac delta functions for each harmonic component. Within this spectrum, the fundamental frequency component we desire is as follows:

(3) $$a_{1}\left(t\right)=sinc\left(\frac{\omega_{out}T}{2}\right)\sin\left(\omega_{out}t-\frac{\omega_{out}T}{2}\right)=a_{1}\sin\left(\omega_{out}t-\phi_{1}\right)$$

In the DDFS system, let the phase quantization step size of the waveform lookup table be Δ. The quantization error due to this step size can be modeled as being uniformly distributed within the interval $\left[-\frac{\Delta }{2},\frac{\Delta }{2}\right]$. The mean square error for such a uniform distribution is given by the following:

(4) $$P_{\mathrm{quant}}=\frac{\Delta^{2}}{12}$$

Since the DDFS system samples the output signal at the clock frequency $f_{\mathrm{clk}}$, the quantization noise power accumulates across the entire sampling bandwidth. Given that noise is generated at each sampling instance, the total quantization noise power is proportional to the clock frequency. Hence, the total quantization noise power is as follows:

(5) $$P_{q}=\frac{\Delta^{2}}{12}\cdot f_{\mathrm{clk}}$$

Quantization noise is not uniformly distributed across the frequency spectrum; instead, it follows a specific pattern. For the sinusoidal output of the DDFS, the quantization noise spectrum, after filtering, primarily concentrates around the fundamental frequency of the signal. This distribution can be described using the sinc function. For the DDFS output signal, the quantization noise in the frequency spectrum is suppressed near the fundamental frequency by the low-pass filter, resulting in the error power $P_{q}$ for traditional DDFS methods:

(6) $$P_{q}=\frac{\Delta^{2}}{12}\cdot f_{clk}\cdot sinc^{2}\left(\frac{\omega_{out}T}{2}\right)$$

### 2.2. FPGA-Based DDFS Architectural Design

**Figure 3:**
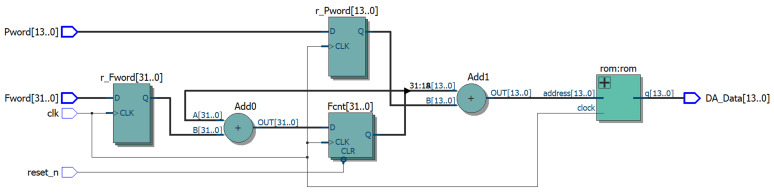
Traditional DDFS architecture RTL.

To achieve high precision, stability, and rapid response, FPGAs are selected for DDFS implementation due to their parallel processing capabilities and configurable resources [17,18,19,20,21]. These features enable flexible adjustments in design parameters, such as the bit widths of the lookup table and phase accumulator. The architecture is implemented using Verilog in the Quartus tool [22], with the Register Transfer Level (RTL) as presented in Figure 3.

In traditional DDFS architectures, the lookup table ROM maps discrete phase values to D-bit wide sine wave amplitudes. Although an infinitely large D value would theoretically ensure error-free phase-to-amplitude conversion, practical FPGA implementations are constrained by ROM resources and DAC performance [4]. Consequently, the D bit width is limited, leading to amplitude errors in the lookup table and spurious components in the DDFS output spectrum [2,23].

## 3. Optimized Design of DDFS Architecture

**Figure 4:**
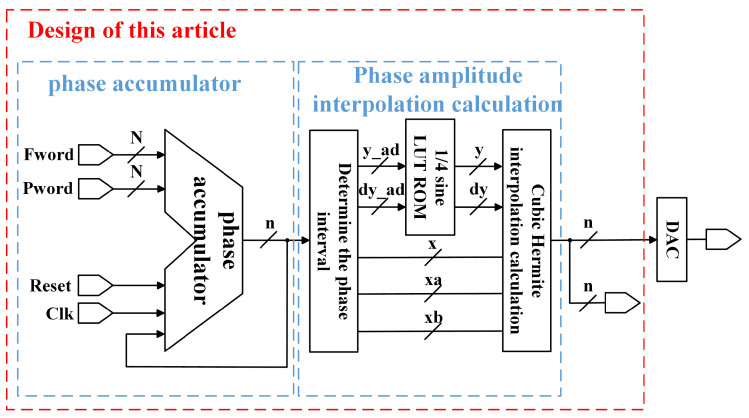
Optimization architecture diagram.

To reduce spurious signals caused by quantization and to enhance the SFDR of the output signal, this study employs a cubic Hermite interpolation algorithm and a single-quadrant storage method to optimize the traditional DDFS architecture. The optimized architecture is shown in Figure 4.

### 3.1. Optimization of Interpolation Algorithm

**Figure 5:**
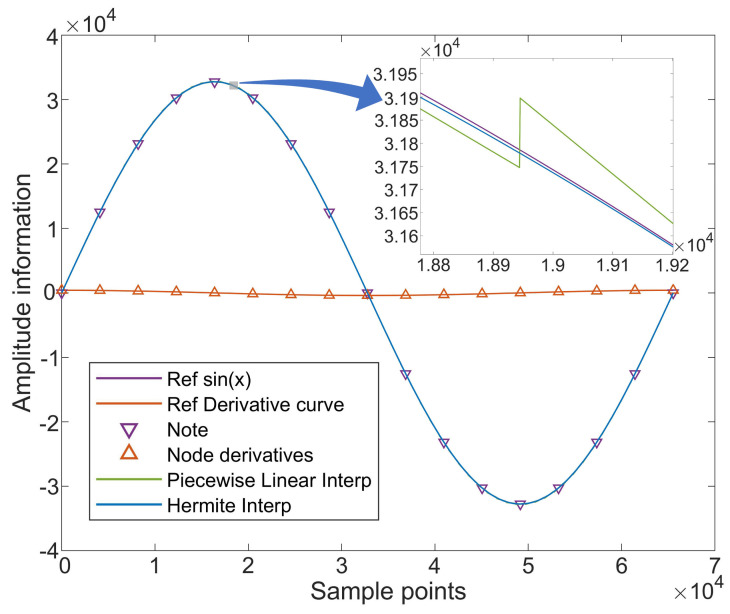
Comparison of two interpolation algorithms.

In the field of signal processing, piecewise linear interpolation algorithms are commonly used due to their simplicity and ease of implementation. However, linear interpolation typically approximates the relationship between adjacent data points by merely connecting them, neglecting the overall continuity of the data distribution. This results in interpolation curves with discontinuous corners and irregular sawtooth patterns, as the model relies solely on local data for predictions, limiting its accuracy based on data density and distribution [2,24,25]. In contrast, cubic Hermite interpolation provides a smoother and more accurate approximation. It not only considers the values at the data points but also includes the first-order derivatives at these points, offering a more coherent and precise depiction of the overall trend. This method is especially effective in processing signals with high-frequency components [17,18,26]. As demonstrated in Figure 5, a comparison of the effects of piecewise linear interpolation and cubic Hermite interpolation on a sine wave signal clearly shows the superior interpolation quality of the cubic Hermite method.

To address amplitude quantization errors in DDFS systems, this paper optimizes the process using the cubic Hermite interpolation algorithm, following these main research steps:

1. Approximate the curve between two sampling points as a cubic polynomial;
2. Specify both function values and first-order derivatives at the two sampling points;
3. Solve for the coefficients of the polynomial;
4. Use the polynomial for interpolation between the two sampling points.

The cubic Hermite interpolation algorithm is detailed by Gibin et al. [18]. The expression for the cubic Hermite interpolation function $I_{h}\left(x\right)$ over the interval [$x_{k}$, $x_{k+1}$] is provided by Equation (7):

(7) $$\begin{array}{c} I_{h}\left(x\right)={\left(\frac{x-x_{k+1}}{x_{k}-x_{k+1}}\right)}^{2}\left(1+2\frac{x-x_{k}}{x_{k+1}-x_{k}}\right)f_{k}+{\left(\frac{x-x_{k}}{x_{k+1}-x_{k}}\right)}^{2}\left(1+2\frac{x-x_{k+1}}{x_{k}-x_{k+1}}\right)f_{k+1}\\ +{\left(\frac{x-x_{k+1}}{x_{k}-x_{k+1}}\right)}^{2}\left(x-x_{k}\right)f_{k}^{\prime }+{\left(\frac{x-x_{k}}{x_{k+1}-x_{k}}\right)}^{2}\left(x-x_{k+1}\right)f_{k+1}^{\prime } \end{array}$$

where $f_{k}$ and $f_{k+1}$ are nodal function values, and $f_{k}^{\prime }$ and $f_{k+1}^{\prime }$ are nodal derivative values.

Direct FPGA implementation of Equation (7) requires a large number of multipliers and dividers, leading to the consumption of substantial FPGA logic units. Therefore, the equation is transformed into a form using basis functions [18], as shown in Equation (8):

(8) $$H\left(x\right)=h_{0}y_{a}+h_{1}y_{b}+h_{2}m_{a}+h_{3}m_{b}$$

where *a*, *b* are the endpoints of the interpolation interval; $y_{a}$ is the signal value at node 1, $m_{a}$ is the signal derivative value at node 1; and $h_{0}$, $h_{1}$, $h_{2}$, and $h_{3}$ are Hermitian basis functions.

(9) $$h_{0}=2t^{3}-3t^{2}+1$$

(10) $$h_{1}=-2t^{3}+3t^{2}=1-h_{0}$$

(11) $$h_{2}=t^{3}-2t^{2}+t$$

(12) $$h_{3}=t^{3}-t^{2}$$

The relative position *t* of the interpolated point *x* between the two nodes is

(13) $$t=\frac{x-x_{a}}{x_{b}-x_{a}}$$

The processed cubic Hermite interpolation algorithm allows for a reduction in the utilization of FPGA logic units, as presented in Table 1.

The error of the cubic Hermite interpolation can be expressed as follows:

(14) $$e_{H}\left(x\right)=f\left(x\right)-f_{H}\left(x\right)$$

Using Taylor expansion, f(x) can be approximated as follows:

(15) $$f\left(x\right)\approx f\left(x_{a}\right)+f^{\prime }\left(x_{a}\right)\left(x-x_{a}\right)+\frac{1}{2}f^{\prime \prime }\left(x_{a}\right){\left(x-x_{a}\right)}^{2}+\cdots$$

The error after cubic Hermite interpolation is dominated by the fourth-order derivative term, which can be further expressed as follows:

(16) $$e_{H}\left(x\right)\approx \frac{f^{\left(4\right)}\left(x\right)}{4!}\cdot {\left(x-x_{a}\right)}^{2}{\left(x-x_{b}\right)}^{2}$$

The error power is the integral mean of the error squared over the entire interval:

(17) $$P_{H}=\frac{1}{T}\int_{x_{a}}^{x_{b}}{\left|e_{H}\left(x\right)\right|}^{2}dx$$

Substituting the formula into Equation (17),

(18) $$P_{H}=\frac{1}{T}\int_{x_{a}}^{x_{b}}{\left(\frac{f^{\left(4\right)}\left(x\right)}{4!}\cdot {\left(x-x_{a}\right)}^{2}{\left(x-x_{b}\right)}^{2}\right)}^{2}dx$$

For a sine wave f(x)=sin(ωx), the fourth-order derivative is as follows:

(19) $$f^{\left(4\right)}\left(x\right)=\omega^{4}\sin\left(\omega x\right)$$

Therefore, the error power can be expressed as follows:

(20) $$P_{H}=\frac{\omega^{8}}{{\left(4!\right)}^{2}T}\int_{x_{a}}^{x_{b}}{\left(x-x_{a}\right)}^{4}{\left(x-x_{b}\right)}^{4}\sin^{2}\left(\omega x\right)dx$$

Comparing the error power $P_{q}$ (Equation (6)) from the traditional method with the error power $P_{H}$ (Equation (20)) after Hermite interpolation reveals the following:

1. The error in the traditional method mainly stems from linear quantization noise. As the quantization step size Δ increases, the error power also increases.
2. The error in Hermite interpolation is due to the approximation error of higher-order derivative terms. The interpolation method reduces the quantization error, particularly in cases of higher resolution and more complex signals, making the effect of the interpolation method more pronounced.

Specifically, in cases of high resolution and complex signals, the error power $P_{H}$ of the Hermite interpolation method is significantly lower than that of traditional methods, demonstrating its superiority in enhancing signal processing accuracy. By reducing quantization noise and improving interpolation precision, the Hermite interpolation method exhibits outstanding performance in high-performance signal processing applications.

### 3.2. Single-Quadrant ROM Table Design

According to the cubic Hermite interpolation formula, calculating the interpolation requires both the function values $f(x_{k})$ at each node $x_{k}$(k=0,1,…,n) and their derivative values $f^{\prime }\left(x_{k}\right)$. This implies that, within a single system clock cycle $f_{clk}$, the optimized architecture must retrieve four values from the ROM. Conventional LUT methods utilize four ROMs to output these values within one clock cycle, significantly increasing ROM resource consumption [9]. To address this, the paper proposes a ROM table design that combines the single-quadrant storage method with the trigonometric conversion relation.

Leveraging the symmetry of sine and cosine waveforms, only the sine sampling values within the range of 0 to π/2 need to be stored, a technique referred to as the single-quadrant storage method [27]. Also, according to the trigonometric relation Equation (21),

(21) $$f^{\prime }\left(x\right)={\left(sinx\right)}^{\prime }=cosx=sin\left(\frac{\pi }{2}-x\right)$$

It can be shown that given a phase value *x*, the value of the derivative of the sine function at that moment in time can be obtained by finding the phase value corresponding to the angle π/2−x in the table. To obtain the magnitude and derivative values of the two nodes simultaneously within a short clock cycle, the optimized architecture uses a dual-port ROM table structure. The ROM table structure comprises *n* sets of 2×M-bit data, where n stands for the number of nodes. The higher *M* bit of the data stores the function value of $f_{k}$(k=0,1,…,n−1), and the lower *M* bit stores the function value $f_{k+1}$(k=1,2,…,n). Moreover, as per the trigonometric function relationship, the derivative value of the node read from the ROM table structure is $f_{k}^{\prime }=f_{m}$(where m=n−k). This structure allows for the efficient retrieval of the function values needed for cubic Hermite interpolation within a shorter clock cycle, significantly improving the ROM’s real-time performance and resource efficiency.

Table 2 compares resource utilization between using and not using the single-quadrant method. While the total number of logical elements slightly increases with the method, due to the overhead it introduces, memory bit usage drops significantly. This suggests that the single-quadrant method significantly optimizes memory usage with minimal impact on logic resources.

### 3.3. Delayed Structural Design

After the phase accumulator obtains the phase information, it is analyzed to extract the address information for the amplitude and derivatives, followed by the execution of the ROM read operation. This process requires two clock cycles: during the first cycle, address decoding is performed, converting the input address into an index for the ROM lookup table. In the second clock cycle, the ROM outputs the data stored at the corresponding index [28].

Since the ROM lookup table requires two clock cycles to read data, the interpolation calculation module will experience a delay of two clock cycles in receiving the node’s amplitude and derivative information. To compensate for this, the optimized architecture incorporates a clock delay module, which employs pipeline delay techniques by adding two delay registers. These registers postpone the node’s phase data, current phase data, and sign information by two clock cycles to maintain synchronization.

### 3.4. Optimized Architecture Logic Design

Based on Equation (8) and the single-quadrant storage method for ROM table design, the traditional DDFS architecture is optimized using cubic Hermite interpolation. The specific steps are as follows:

1. Phase calculation and adjustment.
  The phase of the node is calculated using a phase accumulator. A single-quadrant storage method is employed to perform phase reversal operations and sign changes at specific phase points (1/4, 1/2, and 3/4 cycles) to generate the desired sinusoidal phase data for the cycle.
2. Phase data handling.
  After acquiring the phase data, the key bits (determined by the number of bits in the ROM storage table) are retained, while the remaining bits are cleared to obtain the node data $x_{a}$. The other node data $x_{b}$ are then computed using the sum of $x_{a}$ and the interval width, along with the delayed structure, to ensure synchronized input of parameters for the cubic Hermite interpolation.
3. ROM table structure application.
  A single-quadrant storage method and a dual-port ROM table structure are utilized. Based on the interval node data, the address information for the magnitude and derivative data of the two nodes in the interval are obtained from the ROM.
4. Data read and shift operations.
  Using the obtained address information, the magnitude and derivative data for the two nodes in the interval are read from the ROM. Since each data entry in the ROM table contains information about two nodes in an interval, a shift operation is performed after reading to separately access the data for the two nodes.
5. Cubic Hermite interpolation calculations.
  Based on the parameters derived from the previous steps, the cubic Hermite interpolation algorithm is executed to generate the interpolated waveform data.

**Figure 6:**
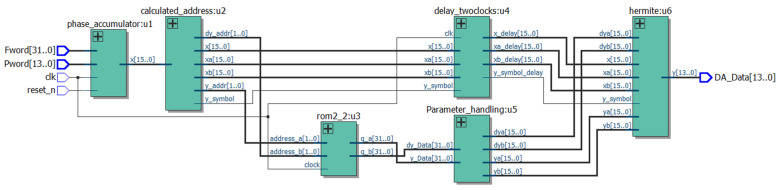
The optimized architecture RTL.

The DDFS optimization architecture RTL based on cubic Hermite interpolation is shown in Figure 6, and Algorithm 1 provides the pseudocode for the cubic Hermite interpolation in DDFS systems.
**Algorithm 1** Cubic Hermite interpolation for DDFS.**Input:** Phase accumulator, ROM //Phase increment and ROM with nodes and derivatives
**Output:** Output waveform value
1:**Main Function: DDFS**2:   Initialize phaseincrement, ROM3:**while** system running **do**4:      phase=phase+phaseincrement5:      adjusted_phase← adjustPhase(phase)6:      $x_{k},f_{k},f_{k}^{\prime }\leftarrow$ ROM[adjusted_phase]7:      $x_{k+1},f_{k+1},f_{k+1}^{\prime }\leftarrow$ ROM[adjusted_phase + 1]8:      waveform_value← sign· cubicHermiteInterpolation(phase, $x_{k}$, $x_{k+1}$, $f_{k}$, $f_{k+1}$, $f_{k}^{\prime }$, $f_{k+1}^{\prime }$)9:      output waveform_value through DAC and LPF10:**end while**11: 12:**Function** cubicHermiteInterpolation(*x*, $x_{k}$, $x_{k+1}$, $f_{k}$, $f_{k+1}$, $f_{k}^{\prime }$, $f_{k+1}^{\prime }$):13:   $t=\frac{x-x_{k}}{x_{k+1}-x_{k}}$ //Calculate the relative position14:   $h_{0}=2t^{3}-3t^{2}+1$ //Hermitian basis function15:   $h_{1}=t^{3}-2t^{2}+t$16:   $h_{2}=-2t^{3}+3t^{2}$17:   $h_{3}=t^{3}-t^{2}$18:   $interpolated\_value=h_{0}\cdot f_{k}+h_{1}\cdot \left(x_{k+1}-x_{k}\right)\cdot f_{k}^{\prime }+h_{2}\cdot f_{k+1}+h_{3}\cdot \left(x_{k+1}-x_{k}\right)\cdot f_{k+1}^{\prime }$19:   **return** interpolated_value20: 21:**Function** adjustPhase(phase) //Adjust phase using single-quadrant storage method22:**if** $phase<\frac{\pi }{2}$ **then**23:        sign=124:        adjusted_phase=phase25:**else if** phase<π **then**26:        sign=127:        adjusted_phase=π−phase28:**else if** $phase<\frac{3\pi }{2}$ **then**29:        sign=−130:        adjusted_phase=phase−π31:**else**32:        sign=−133:        adjusted_phase=2π−phase34:**end if**35:**return** adjusted_phase,sign


## 4. Experiment and Analysis

To verify the effectiveness of the DDFS optimization architecture based on the cubic Hermite interpolation algorithm proposed in this paper, validation was conducted from both simulation and experimental perspectives. The parameter settings for the optimized architecture were identical to those of the traditional DDFS, as shown in Table 3.

### 4.1. ModelSim Software Simulation

The proposed architecture was designed on a Windows 10 operating system using Quartus II 15.0, with waveform simulations performed in ModelSim SE, and the simulation data processed in MATLAB. During the simulation code design, the frequency control word (Fword = 268,740,399) and the phase control word (Pword = 0) were initialized to achieve an output frequency of 6,257,100 Hz. The following outlines the specific steps for implementing co-simulation with ModelSim and MATLAB.

**Figure 7:**
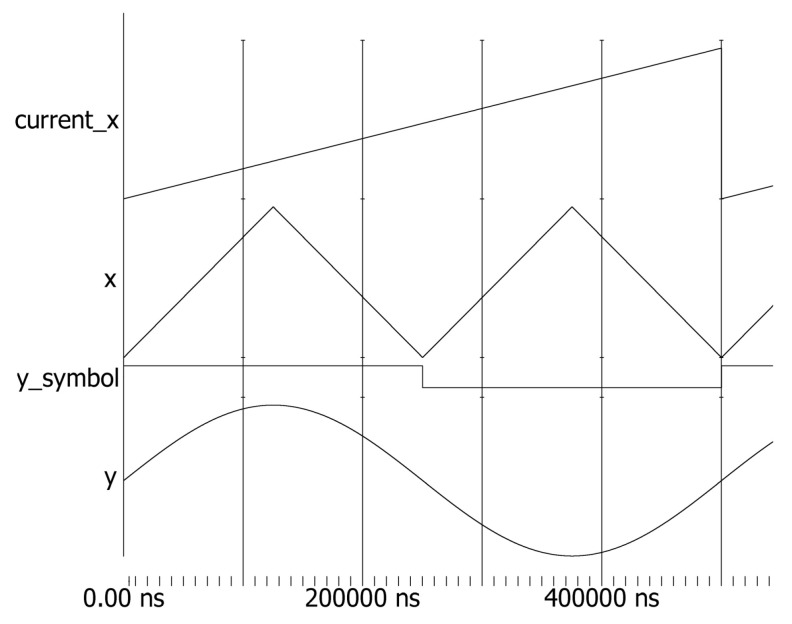
ModelSim simulation.

Initially, a DDFS hardware architecture optimized with the cubic Hermite interpolation algorithm was designed using Verilog. ModelSim simulation tools were then utilized to load test waveforms. The generated sinusoidal analog outputs, as shown in Figure 7, demonstrate that the optimized architecture, based on the cubic Hermite interpolation algorithm, can achieve high-quality waveform reconstruction with smooth signal transitions.

In Figure 7, current_x refers to values obtained from the phase accumulator output, while x represents processed phase information. This involves dividing one period of current_x into four equal parts and starting an inverse operation at each 1/4 period marker. In this architecture, x also serves as the address information, used for forward and reverse reading of amplitude information from the ROM lookup table. The y_symbol is the sign bit, which is set to 0 when the phase is in the third or fourth quadrant. After cubic Hermite interpolation calculations and determining whether to invert the signal based on the sign bit, the final output, y, is produced, ensuring smooth waveform output.

To analyze the spectral information of the simulated waveforms, MATLAB was used to process the waveform files. Initially, in the FPGA simulation code, the $fdisplay() function was utilized for each clock cycle to log the simulated waveform data. After running the ModelSim simulation tool, waveform data for both the optimized cubic Hermite interpolation and traditional DDFS were obtained. These files were imported into MATLAB using the fopen() function to extract the amplitude of the time-domain waveforms. The Blackman window function was applied to process the data, reducing spectral leakage. A Fast Fourier Transform (FFT) was then performed, and a compensation factor for the window function was applied to obtain frequency domain information. Finally, spurious peak search operations were conducted to determine the maximum spurious amplitude and calculate the amplitude–frequency characteristics.

**Figure 8:**
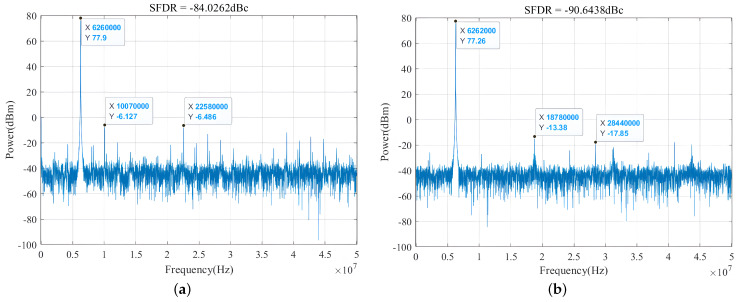
MATLAB calculation of the amplitude–frequency curve through the ModelSim simulation data. (**a**) represents the amplitude–frequency curve of the traditional DDFS, and (**b**) represents the amplitude–frequency curve after the cubic Hermite interpolation optimization.

The spectral analysis results of the simulated waveforms are shown in Figure 8. In Figure 8a, the amplitude–frequency curve of the traditional DDFS architecture is displayed. The main frequency amplitude reaches 77.9 dB, while the sub-maximum spurious frequency amplitude is −6.486 dB. Further calculations reveal that the SFDR of the traditional architecture is −84.0262 dBc. Figure 8b presents the amplitude–frequency curve after applying cubic Hermite interpolation optimization. The main frequency amplitude of the optimized architecture is 77.26 dB, and the sub-maximum spurious frequency amplitude is −13.38 dB.

In Figure 8a, the traditional DDFS exhibits prominent spurious components at frequencies such as 10.07 MHz and 22.58 MHz, which result from deviations in stored values caused by finite word lengths and quantization within the ROM table, introducing quantization noise and degrading spectral purity. In contrast, Figure 8b shows a reduction in spurious components around 18.78 MHz and 28.44 MHz, demonstrating that the optimized architecture effectively reduces quantization noise and suppresses spurious components. Additionally, the SFDR of the optimized DDFS system reaches −90.6438 dBc, an improvement of 6 dBc over the traditional DDFS, indicating an expanded dynamic range.

### 4.2. FPGA Platform Experiment

**Figure 9:**
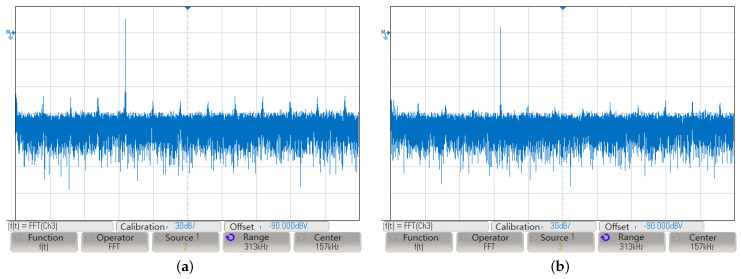
Measured oscilloscope output results; (**a**) is the traditional DDFS, and (**b**) is the method proposed in this paper.

The experimental test platform comprises a DC power supply, an FPGA-based DDFS frequency synthesizer, an oscilloscope, and coaxial cables. The FPGA module used is the Altera FPGA Cyclone II EP4CE10F17C8 chip, where digital logic circuits such as the phase accumulator and cubic Hermite interpolation module were designed and implemented. The DAC module utilizes the Texas Instruments 14-bit parallel input DAC chip, DAC904, which supports an update rate exceeding 165 MSPS, ensuring optimal dynamic performance for this experiment. A low-pass filter module was employed to remove noise and interference from the DAC output waveform. The Keysight MSO-X3054A oscilloscope was selected to measure spurious suppression performance. Figure 9a illustrates the amplitude–frequency curve of the traditional DDFS method, while Figure 9b shows the curve after applying the cubic Hermite interpolation algorithm.

From Figure 9, it is calculated that the SFDR of the DDFS system optimized by the cubic Hermite interpolation is −88.134dBc, and the SFDR of the traditional DDFS is −81.961 dBc. As seen in Figure 9b, some residual spurious components remain, which can be attributed to the limitations of cubic Hermite interpolation in capturing higher-order terms. Nevertheless, compared to the traditional method, the interpolation approach significantly reduces errors. The noticeable improvement in the noise floor and the reduction in spurious signals in the spectrum further confirm this enhancement.

**Figure 10:**
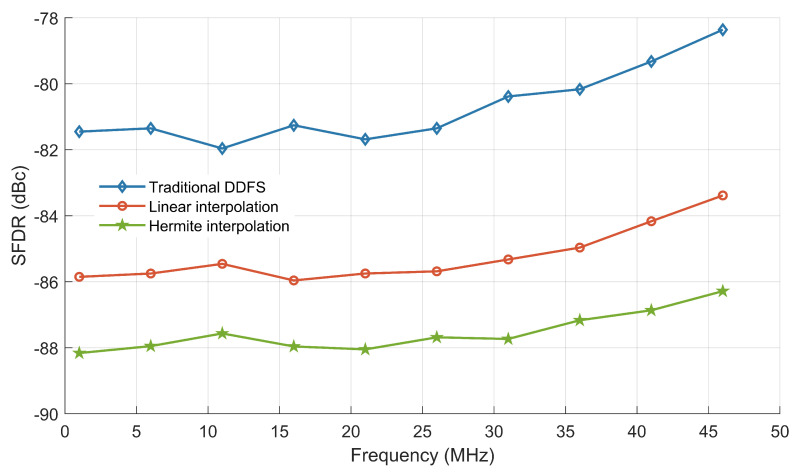
SFDR performance of different output frequencies.

To evaluate the performance of the optimized scheme across different operating frequencies, several experiments were conducted. As shown in Figure 10, the traditional DDFS architecture displays fluctuating SFDR values, ranging from approximately −82 dBc to −84 dBc, indicating inconsistent spectral purity. In contrast, the linear interpolation-based scheme exhibits more stable SFDR performance, maintaining around −86 dBc. However, the cubic Hermite interpolation method achieves the best results, with SFDR values consistently around −88 dBc across the frequency range, reflecting a significant improvement in spectral purity.

The analysis also highlights some discrepancies between the FPGA platform experiment results and the ModelSim simulation. These differences are likely due to the inability of the simulation model to fully replicate the effects of actual FPGA internal signals, such as timing hazards and LUT mismatches. Moreover, parasitic parameters of FPGA pins and PCB wiring may cause signal distortion and attenuation, increasing non-harmonic components and resulting in larger measurement errors.

**Figure 11:**
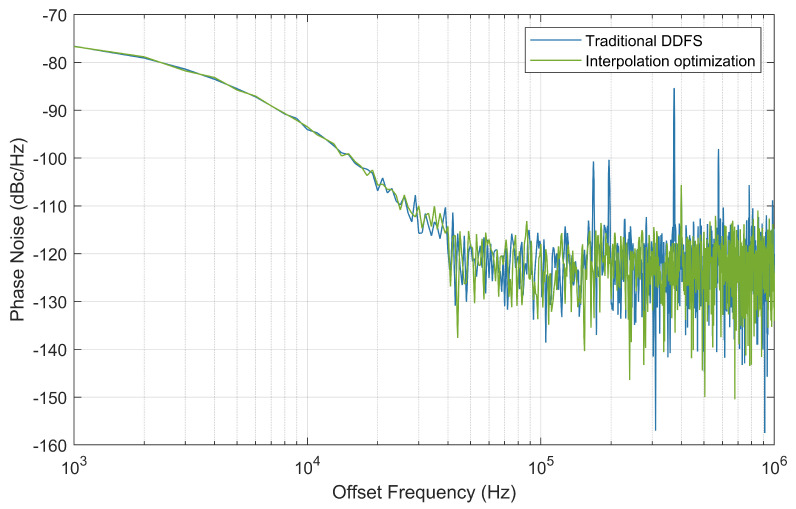
Single sideband phase noise.

Figure 11 shows the phase noise characteristics of the traditional DDFS compared to the interpolation-optimized DDFS. Both designs exhibit similar phase noise behavior at lower offset frequencies, with the noise floor decreasing as the offset frequency increases. However, beyond 20 kHz, the interpolation-optimized DDFS demonstrates a clear improvement over the traditional design, particularly in the higher offset frequency range, where it maintains a lower noise floor. This result emphasizes the effectiveness of the proposed interpolation technique in minimizing phase noise.

Table 4 compares the proposed design with several recent DDFS implementations that achieve high-quality SFDR performance. In earlier DDFS studies, the highest SFDR recorded was 74 dBc using the second-order parabolic equation, the new CORDIC SFDR value is 72.2 dB. More recent studies employing impulse DDFS, while structurally simpler, achieved only 41 dBc. In contrast, the proposed optimized design attains an SFDR of 88.134 dBc, significantly outperforming other DDFS designs, whether FPGA-based or simulated.

Referring to Table 5, the resource utilization of the four methods is compared. It can be calculated that the total ROM compression ratio of the method proposed in this article is 1792:1, which is significantly higher than the previous best value of 843:1 [15]. This demonstrates that, in addition to achieving better SFDR performance, the method also achieves a superior resource compression ratio. However, it should be noted that this method also occupies a large quantity of logic resources and multipliers.

## 5. Conclusions

This paper outlines the fundamental principles of traditional DDFS systems, identifying key issues such as excessive ROM resource consumption and limited SFDR performance. To address these challenges, an optimized DDFS architecture based on cubic Hermite interpolation is proposed. The experimental results demonstrate the effectiveness of this approach in reducing ROM usage and improving SFDR, as it significantly mitigates spurious interference caused by amplitude quantization during the DDFS digitalization process. This presents a promising new strategy for designing high-precision frequency sources with a wide dynamic range.

While the proposed method shows clear advantages in ROM resource compression and SFDR enhancement, further improvements are possible. Specifically, optimizing the algorithm’s implementation could help reduce FPGA logic resource usage. Such refinements will broaden the applicability of this optimized DDFS scheme, making it better suited for systems that require high-performance frequency sources, such as radar and communication systems.

## Figures and Tables

**Table 1:** Resource utilization.

Logic Unit	Equation (7)	Optimized
Adder	19	11
Multiplier	14	11
Defaulter	6	1

**Table 2:** Comparison of resource utilization.

	Total Logic Elements	Total Memory Bits
Using the single-quadrant method	1394	128
Single-quadrant method not used	1326	507

**Table 3:** Parameter settings.

Parameter Name	Set Value
$f_{clk}$	100 MHz
Fword	32 bit
Pword	14 bit
DAC	14 bit

**Table 4:** SFDR performance comparison.

	[5] TVLSI	[4] JSPS	[3] ACCESS	[29] EWDTS	[13] TVLSI	This Work
Year	2018	2019	2020	2020	2021	2023
Process (nm CMOS)	180	FPGA	65	FPGA	45	FPGA
$F_{\mathrm{word}}$ (bits)	32	32	32	32	25	32
Output resolution (bits)	24	24	9	16	25	14
Clock rate (MHz)	71.9	100	2000	251	2000	100
Verification	Mears.	Mears.	Sim.	Mears.	Sim.	Mears.
SFDR (dBc)	74	68.4242	70.8	72.2	41	88.134

Sim. = Simulation, Meas. = Measurement

**Table 5:** Resource utilization comparison of four methods.

Resource Utilization Rate	Traditional Method	Piecewise Linear Approximation Method [2]	Pulse Width Modulation Method [11]	Proposed Method
Device	EP4CE10F17C8	EP1C12Q24017	EP4CE55F23C6	EP4CE10F17C8
Total logic elements	94/10,320 (<1%)	1104/12,060 (9%)	36,011/55,856 (64%)	1394/10,320 (14%)
Total memory bits	229,376/423,936 (54%)	180,275/23,9616 (75%)	1,286,144/2,396,160 (54%)	128/423,936 (<1%)
Total pins	19/180 (11%)	19/173(11%)	181/325 (56%)	19/180 (11%)
Embedded multiplier 9-bit elements	0/46 (0%)	N/A	184/308 (60%)	46/46 (100%)
Total PLLs	1/2 (50%)	1/2 (50%)	2/4 (50%)	1/2 (50%)

## Data Availability

The data used to support the findings of this study are available from the corresponding author upon request. The data are not publicly available due to privacy.
